# The impact of networks on clinical trials in the United Kingdom

**DOI:** 10.1186/1745-6215-10-100

**Published:** 2009-11-04

**Authors:** Sze May Ng, Alan Michael Weindling

**Affiliations:** 1 School of Reproductive and Developmental Medicine, University of Liverpool, Liverpool, UK

## Abstract

The conduct of clinical trials in the UK has been affected by the recent introduction of managed clinical networks, clinical research networks and rigorous governance regulations. This commentary considers the challenges that these changes have posed for clinical triallists in the UK, based on experiences derived in the conduct of a multicentre neonatal clinical trial under the conditions that now prevail. We conclude that the considerable skills and knowledge that are now required to be an effective Principal Investigator should be recognised and that application processes, including issuing honorary contracts, should be simplified and centralised.

## Introduction

A recent paper by Vickers and Scardino in *Trials *[1] discussed the barriers facing patient accrual into cancer trials, focussing particularly on finance and regulatory issues and the heterogeneity of cancer patients. Similar challenges to performing clinical trials in the United States were further described by Crawford and Tangen[2]. In this commentary, we draw attention to three changes, which have recently impacted on the management and conduct of clinical trials in general: (a) the formation of managed clinical networks (MCNs), (b) clinical research networks (CRNs); these major organisational changes coincided with a third, (c) the introduction of rigorous governance regulations. An unanticipated consequence of these three developments was an increase in the complexity involved in setting up and conducting clinical trials in the UK. This complexity is difficult enough for experienced triallists and the practical nature of clinical research is that successful trials need to be undertaken in a clinical environment and can require the involvement of district general hospital clinical specialists We have drawn on our experiences with a different group of patients, newborn babies, which shares some of the issues identified by Vickers and Scardino, but highlights other factors, which were not anticipated but which are relevant to all patient groups and which are likely to affect other clinical trials in the UK. These interdependent developments are described more fully below with some suggestions for improving the process.

### The Effect of Managed Clinical Networks (MCNs) on Clinical Trials

These networks of health staff and organisations, ranging from primary to tertiary health care, are intended to ensure that high quality clinically effective services are fairly distributed and that health costs are reduced[3]. Their introduction raised a number of issues.

By developing as a MCN, a service, which is accessed locally, can make a range of sub-specialties available to all and so improve the quality of patient care. Over the years, more and more sub-specialties across the UK have developed MCNs, ranging from coronary heart disease, stroke and diabetes. Neonatal MCNs were established across England in 2004[4]. Against a backdrop of the tremendous human cost and financial burden of preterm birth and its consequences, estimated at an annual cost of £939 million to the UK[5], they were intended to provide a structure and organisation for highly specialised perinatal care, allowing equitable access for all very sick infants with the intention of improving the quality of their care generally. This resulted in the designation of regional hospitals for the care of the sickest and most immature infants, sometimes at considerable distance from the local hospital where their mothers had booked for obstetric care. Such babies are then transferred back to their local maternity hospitals when clinically stable.

From a clinical trial perspective, the specialist therapy that is being tested is often only available at the regional centre. This happened in several successful neonatal trials, e.g. the TOBY study of total body hypothermia for asphyxiated term babies [6] and the UK study of extracorporeal membrane oxygenation[7], as well as in the TIPIT trial[8], a randomised controlled trial where thyroxine supplementation was given to babies born below 28 weeks' gestation to test the hypothesis that such supplementation improves brain growth. Recruitment to TIPIT took place at five regional recruiting centres during the first five days of life. Recruited babies, whose mothers had booked elsewhere for their maternity care, were then transferred back to 24 other non-recruiting local hospitals within the MCN, known in clinical trial jargon as "step-down sites". TIPIT, like other such studies, involved later trial-specific assessments and continuation of the trial intervention at the local hospitals. The regulatory processes for this are complex and considered below.

### Effect of Clinical Research Networks (CRNs) on clinical trials

CRNs were developed to support and coordinate high quality clinical research and to facilitate the conduct of clinical trials and other studies within the National Health Service (NHS) [9]. The National Institute for Health Research (NIHR) Comprehensive Clinical Research Network (CCRN) was created as part of the government's research and development strategy, "Best Research for Best Health" to provide a world-class infrastructure for clinical trials in all areas of disease and clinical need within the NHS[9]. The NIHR CCRN works together with the six Topic Specific CRNs and a Primary Care Research Network to support a national portfolio of clinical trials and other studies. The network that we had most involvement with was the Medicines for Children Research Network (MCRN). The MCRN was established in 2006 to improve the clinical research environment for children with a stated aim "to facilitate the conduct of randomised prospective trials and other well-designed studies of medicines for children, including those for prevention, diagnosis and treatment" [10]. A similar function is provided by CRN for other patient groups [9].

If a trial involves an unlicensed medicine without support by a large pharmaceutical company, considerable resources are required. There needs to be intensive input from trust pharmacists and trust R&D (Research and Development) departments to deal with manufacturing the investigational medicinal product (IMP), its quality control, blinding and dispensing. This requires considerable specialist knowledge and resources. Furthermore, a clinical medicinal trial requires sponsors, usually a university and an NHS trust, who are responsible for its design, conduct, recording and reporting.

TIPIT was the first clinical trial to benefit from the MCRN since its establishment in 2006[8]. The catchment area for the main hospital for TIPIT included several NHS regions and managed clinical networks to which recruited babies were transferred back. Several local MCRN research networks provided invaluable assistance with site initiations, gaining separate ethical and R&D approvals and honorary contracts for investigators in all 29 trusts involved in the trial. Nursing staff employed by the MCRN also assisted in trial promotion to relevant nursing, medical and R&D staff and provided some pharmacy support. The MCRN also assisted sponsors in the monitoring of GCP compliance during the conduct of the trial and supported trust pharmacies and TIPIT successfully underwent three MHRA inspections.

### Regulation of clinical trials and step-down sites

At the same time as the networks described above were being set up, a rigorous regulatory framework was established in the UK. The legal framework for conducting clinical trials was prepared under Directive 2001/20/EC, the European Union Clinical Trials Directive (EUCTD) [11]. The EUCTD established specific provisions for the conduct of clinical trials and implementation of Good Clinical Practice (GCP)[12]. The EUCTD was transposed into UK law as the Medicines for Human Use (Clinical Trials) Regulations on the 1^st ^May 2004 (SI 2004/1031) [13]. The Medicines for Human Use (Clinical Trial) Amendment Regulations (SI 2006/1928) came into force on 29 August 2006 to incorporate the GCP Directives[14]. In the UK, all clinical trials involving medicinal products must now include stringent internal regulation and adhere closely to these guidelines.

These changes, which were instituted between 2004 and 2006, were aimed at promoting the scientific value of clinical trials. However, they have increased enormously the complexities. The impact of MCNs on the conduct of trials has been significant particularly in terms of legislative regulation. Now all step-down sites within a MCN require separate ethical and R&D approval even if no patients from that hospital are actually recruited into the trial.

Our own experience was that since babies were not infrequently transferred from one neonatal network to another, sometimes a hundred miles away or more, it was not possible to predict all the hospitals from where potential trial subjects might come. As there was no centralised system for approval by individual trusts' R&D committees, we had to apply directly to every trust R&D committee within and outside the region, guessing at the likelihood of a baby being transferred to that hospital. The use of step-down sites gave rise to particularly complex arrangements. In TIPIT's case, each trust had to apply to the National Research Ethics Service (NRES)[15] for full Site Specific Assessment (SSA) Approval for trial medications to be continued and follow up assessments to be done. (Under the complicated terms of NRES approvals, the final trial assessment does not require a SSA if the trial intervention is not required, but application for SSA exemption must still be approved by NRES. In addition, individual step down sites require separate approval by their own R&D committees. These are discussed further below.)

One way that the national system has progressed is through the establishment of a coordinated system for gaining approval for taking part in a clinical trial. Previously, each hospital authority had to be approached individually for ethical approval. This has been replaced by a more streamlined system. The NIHR Coordinated System for gaining trial approvals was set up in November 2008 and incorporated two regulatory agencies, the Medicines and Healthcare Products Regulatory Agency (MHRA)[14] and NRES[15].

In an attempt to simplify the application process for approval for a clinical trial, an Integrated Research Application System (IRAS)[16] was established to capture all relevant information in a single application process. Although IRAS provides a single ethical committee application process, there is still no centralised system for approval by trust R&D committees and multi-centred trials still require separate approvals from individual trusts. Approval of clinical trials by individual R&D departments in the UK varies considerably and is generally slow: two recent studies reported a median delay of 44 days[17,18]: only half the R&D departments surveyed used efficient methods such as online R&D application forms.

The status of each site needs to be specified. If recruitment, the trial intervention and follow-up are to be undertaken on that site, full approval is required and a Principal Investigator (PI) is needed. The PI may not have been involved in the conception of the study but is required to have a deep knowledge of the protocol, be available for 24 hour adverse event reporting, and needs to be trained in GCP [12]. Alternatively, if only the trial's stated follow-up aspects of care are to be undertaken, the specified site may apply for SSA exemption and a 'Named Clinician' needs to be identified as the responsible person responsible for the study at that site. This individual is responsible for the study at the site and usually a NHS clinician.

Most PIs are at present unlikely to see taking part in trials as their main role. Particularly at step-down sites, our experience was that some clinicians were so daunted that they declined to even start the process. This was in keeping with the observations of other triallists, who have reported that the impact of administrative burdens imposed in UK to the EU Clinical Trials Directive even resulted in the failure of some trials[19,20]. This is clearly not in the spirit of promoting a sound evidence base for clinical practice when the existing evidence is insufficient. The observation leads us to the suggestion that, because of the regulatory complexities and the importance of the establishment of a sound evidence base for clinical practice, one of each group of clinicians takes on the role of lead investigator for all trials that are done on patients in that department. This approach would be analogous to the development of specific expertise in either management or teaching, which are recognised for clinical excellence awards.

Participating trusts also require honorary contracts for all research personnel. To simplify the process, the NIHR established honorary research passports [21], but these are only accepted at the discretion of individual trusts and still have to be submitted individually to each trust's human resource department. In the TIPIT study, 24 out of the 29 trusts within the MCN did not accept the standard research passport and required their own individual application and assessment involving a detailed occupational health assessment prior to issuing the honorary contracts. Other researchers have also reported that they were required to obtain unnecessary honorary contracts and Criminal Record Bureau checks during the process of obtaining R&D approvals[22,23]. It would be enormously helpful if the proposed "research passports", piloted in Manchester and promoted by the R&D forum and UKCRN, were adopted by all trusts.

## Discussion

The effects of these three recent developments on the conduct of clinical trials were experienced to full effect in the course of the establishment and management of TIPIT [8], which was entirely funded through a Medical Research Council fellowship. This fellowship had the primary aim of providing training for an individual in research techniques and the largest part of the grant was for salary with a relatively small amount available for running costs (£10,000 per annum). However, for training to be effective, the study that the research fellow is involved in needs to be worthwhile and robust. In this case, the research fellow was to gain the competences required to establish and run a high quality multi-centre randomised controlled trial and to analyse its complex results. There was therefore heavy reliance on NHS resources and those of the UK's developing MCN and CRN infrastructure. This combination resulted in a successful trial, which completed recruitment ahead of its predicted time at a low cost to the MRC.

**Figure 1:**
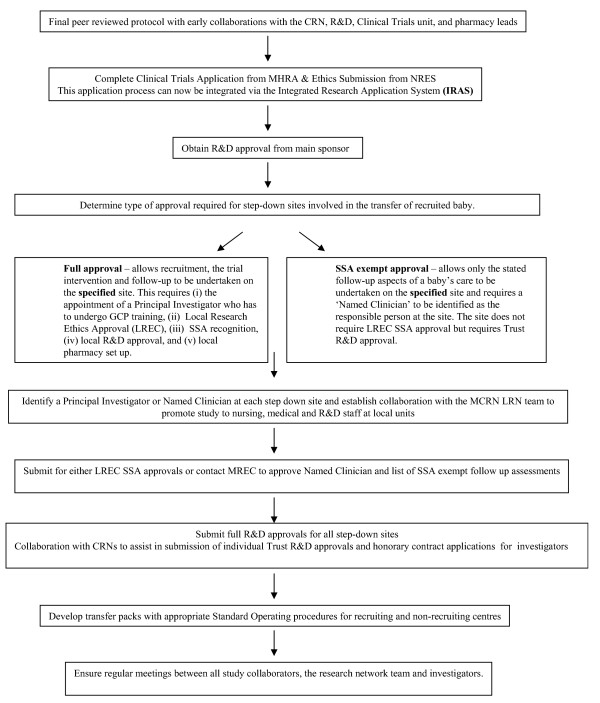
**Flowchart for setting up and conducting a multi centred clinical trial**.

Although modern legislation has improved the scientific rigour of such studies, it has also added to their intricacy. MCNs, while improving the effectiveness of clinical care, have added to the complexities of running an effective clinical trial. It is clear that meeting all the new regulatory requirements and taking account of the effect of managed clinical networks (particularly step-down units) on clinical trials is outside the present experience of even experienced triallists. However, CRNs have improved the cost effectiveness of trials through the provision of an established, separately funded network, which is able to assist in patient accrual, promotion of the trial to nursing, medical and R&D staff and in the trial's day to day management. Our experience of a publicly-funded trial with a limited budget and limited staff was that the MCRN's support role was crucial. The process of setting up and conducting a multicentred clinical trial under the current regulatory system is described in Figure 1.

## Conclusion

We conclude with several recommendations. Firstly, that there should be further development of the infrastructure to support clinical trials within the framework of MCNs and CRNs. This should aim to achieve governance in a timely and effective manner, but with less bureaucracy. This could be achieved by the acceptance by each trust of a common process. Secondly, triallists need to establish collaborations with relevant departments and agencies early in planning, which should include the relevant research and clinical networks. We would urge clinical research networks to provide more support with the bureaucracy associated with the establishment and progression of a clinical trial. Thirdly, application processes, including the provision of honorary contracts, should be simplified and centralised. Finally, the competences required to be an effective PI should be given managerial recognition.

## Abbreviations

(MCNs): Managed clinical networks; (CRNs): Clinical research networks; (NIHR): National Institute for Health Research; (CCRN): Comprehensive Clinical Research Network; (MCRN): Medicines for Children Research Network; (R&D): Research and Development; (IMP): Investigational medicinal product; (NRES): National Research Ethics Service; (SSA): Site Specific Assessment; (MHRA): Medicines and Healthcare Products Regulatory Agency; (IRAS): Integrated Research Application System; (GCP): Good Clinical Practice; (PI): Principal Investigator.

## Competing interests

The authors declare that they have no competing interests.

## Authors' contributions

SMN and AMW conceived the concepts in the manuscript. SMN wrote the first draft. Both authors revised the manuscript and approved the final manuscript.

## References

[B1] Vickers AJ, Scardino PT (2009). The clinically-integrated randomized trial: proposed novel method for conducting large trials at low cost. Trials.

[B2] Crawford ED, Tangen CM (2009). Clinical trials: 'Clinical integration': laudable, but challenging. Nat Rev Urol.

[B3] Clark JD (2007). Managed clinical networks: what are they and how do they work?. Dent Update.

[B4] Marlow N, Bryan Gill A (2007). Establishing neonatal networks: the reality. Arch Dis Child Fetal Neonatal Ed.

[B5] Wolke D, Samara M, Bracewell M, Marlow N (2008). Specific language difficulties and school achievement in children born at 25 weeks of gestation or less. J Pediatr.

[B6] Shankaran S, Pappas A, Laptook AR, McDonald SA, Ehrenkranz RA, Tyson JE, Walsh M, Goldberg RN, Higgins RD, Das A (2008). Outcomes of safety and effectiveness in a multicenter randomized, controlled trial of whole-body hypothermia for neonatal hypoxic-ischemic encephalopathy. Pediatrics.

[B7] Bennett CC, Johnson A, Field DJ, Elbourne D (2001). UK collaborative randomised trial of neonatal extracorporeal membrane oxygenation: follow-up to age 4 years. Lancet.

[B8] Ng SM, Turner MA, Gamble C, Didi M, Victor S, Weindling AM (2008). TIPIT: A Randomised Controlled Trial of Thyroxine in Preterm Infants under 28 weeks' Gestation. Trials.

[B9] UK Clinical Research Network. http://www.ukcrn.org.uk/index.html.

[B10] Medicines for Children Research Network. http://mcrn.org.uk/.

[B11] (2001). EU Clinical Trials Directive. http://www.wctn.org.uk/downloads/EU_Directive/Directive.pdf.

[B12] Good Clinical Practice. http://www.mhra.gov.uk/Howweregulate/Medicines/Inspectionandstandards/GoodClinicalPractice/index.htm.

[B13] UK Medicines for Human Use (Clinical Trials) Regulations. http://www.uk-legislation.hmso.gov.uk/si/si2004/20041031.htm.

[B14] Medicines and Healthcare products Regulatory Agency. http://www.mhra.gov.uk/index.htm.

[B15] NHS Research Ethics Service. https://www.nresform.org.uk/AppForm/display/login.asp?b=1.

[B16] (2008). Integrated Research Application System (IRAS). https://www.myresearchproject.org.uk/SignIn.aspx.

[B17] Al-Shahi Salman R, Brock TM, Dennis MS, Sandercock PA, White PM, Warlow C (2007). Research governance impediments to clinical trials: a retrospective survey. J R Soc Med.

[B18] Hackshaw A, Farrant H, Bulley S, Seckl MJ, Ledermann JA (2008). Setting up non-commercial clinical trials takes too long in the UK: findings from a prospective study. J R Soc Med.

[B19] Hanning CD, Rentowl P (2006). Harmful impact of EU clinical trials directive: trial of alerting drug in fibromyalgia has had to be abandoned. Bmj.

[B20] Watson M (2006). Harmful impact of EU clinical trials directive: and so has trial of melatonin in cancer related weight loss. Bmj.

[B21] (2006). National Institute for Health Research. http://www.nihr.ac.uk/.

[B22] Galbraith N, Hawley C, De-Souza V (2006). Research governance: research governance approval is putting people off research. Bmj.

[B23] Elwyn G, Seagrove A, Thorne K, Cheung WY (2005). Ethics and research governance in a multicentre study: add 150 days to your study protocol. Bmj.

